# Local and systemic XAGE-1b-specific immunity in patients with lung adenocarcinoma

**DOI:** 10.1007/s00262-015-1716-2

**Published:** 2015-05-30

**Authors:** Mehrdad Talebian Yazdi, Nikki M. Loof, Kees L. M. C. Franken, Christian Taube, Jaap Oostendorp, Pieter S. Hiemstra, Marij J. P. Welters, Sjoerd H. van der Burg

**Affiliations:** 1 Department of Pulmonology, Leiden University Medical Center, Leiden, The Netherlands; 2 Department of Clinical Oncology, Leiden University Medical Center, Building 1, K1-P, PO box 9600, 2300 RC Leiden, The Netherlands; 3 Department of Infectious Diseases, Leiden University Medical Center, Leiden, The Netherlands; 4 Department of Clinical Pharmacy and Toxicology, Leiden University Medical Center, Leiden, The Netherlands

**Keywords:** XAGE-1b, CT antigen, Adenocarcinoma, Lung cancer

## Abstract

XAGE-1b is a cancer/testis antigen aberrantly expressed in pulmonary adenocarcinoma. Systemic antibody and T cell responses have been demonstrated in adenocarcinoma patients, but so far, local antigen-specific immunity has not been reported. In this study, XAGE-1b expression by tumor cells as well as the presence of systemic and/or local XAGE-1b-specific immunity was assessed in peripheral blood, tumor tissue and tumor-draining lymph nodes of Caucasian patients with pulmonary adenocarcinoma. XAGE-1b protein expression was detected in 43.6 % (17 of 39) of patients when at least two different parts of a resected tumor were assessed. In 20 patients, analysis of T cells isolated and expanded from the primary tumor and its draining lymph node demonstrated XAGE-1b-specific responses in two patients. XAGE-1b-specific immunoglobulin G antibodies were found in 3 of 40 patients. These three antibody-positive patients had also mounted a systemic T cell response to XAGE-1b, measured by proliferation, cytokine production and expression of T cell activation markers on peripheral blood mononuclear cells. The population of XAGE-1b-specific T cells comprised both CD4+ and CD8+ T cells secreting both type I and II cytokines. Epitope mapping showed that T cells predominantly targeted the N-terminal part of the XAGE-1b protein, while the B cell response was directed against the C-terminal domain. Our study for the first time provides evidence for the presence of XAGE-1b-specific T cells within adenocarcinoma tissue, which supports the concept that XAGE-1b acts as a genuine tumor antigen and, therefore, might form an attractive target for a vaccine-based approach of immunotherapy.

**Electronic supplementary material:**

The online version of this article (doi:10.1007/s00262-015-1716-2) contains supplementary material, which is available to authorized users.

## Introduction

Lung cancer is the most common cause of cancer mortality in men in the developed world and one of the leading causes in women [1]. Non-small cell lung cancer (NSCLC) comprises about 80 % of all lung cancers [2]. The 5-year survival rates rapidly drops with increased stage at diagnosis [3]. The current treatment modalities include surgery, radiotherapy combined with chemotherapy or palliative chemotherapy [4]. Active immunotherapy, focusing on the reinforcement of the tumor-specific T cell response, has emerged as a new modality to treat cancer [5]. NSCLC is characterized by infiltration of different types of immune cells. Infiltration with M1 macrophages and T cells is positively associated with clinical outcomes, suggesting a protective role for the immune system in NSCLC [6]. This is supported by the recent finding that infusion of antibodies blocking programmed cell death protein 1 (PD1) on T cells has clinical impact in advanced NSCLC [7]. Peptide-based therapeutic vaccines aim at the induction of tumor-specific T cell responses [5]. This approach is highly dependent on the identification of suitable tumor antigens [8]. An important group of tumor antigens is encoded by the cancer/testis (CT) genes. These CT antigens are present in a significant subset of tumors, including NSCLC [9], and comprise XAGE-1. The XAGE-1 protein has four transcripts (a, b, c and d), of which XAGE-1b (81 amino acids) is the mainly expressed isoform [10, 11]. Nuclear staining has been observed in 53 % of pulmonary adenocarcinomas, a subtype that accounts for 40 % of NSCLC, but not in adjacent normal tissues indicating its preferential expression by cancer cells [12]. A positive association between the expression of XAGE-1b and HLA class I with prolonged survival was reported [10], although no link with XAGE-1b-specific immunity was made. A recent study revealed the presence of XAGE-1b-specific antibodies in 10 % of all NSCLC patients and in 19 % of stage IIIb/IV adenocarcinoma patients. More than half of the patients with a XAGE-1b antibody response displayed a concomitant systemic CD4+ and CD8+ T cell response [13].

To date, studies on XAGE-1b have been performed in Asian populations but not in Caucasian subjects. Furthermore, no data exist on the presence of XAGE-1b-specific T cells within the tumor or its draining lymph node. To this end, we have conducted an explorative study in which a European cohort of patients with pulmonary adenocarcinoma was studied with respect to XAGE-1b expression and the presence of systemic and local XAGE-1b-mediated immunity.

## Materials and methods

### Patients and tissue collection

Forty patients with histologically proven primary NSCLC, subtype adenocarcinoma, were included from 2011 to 2014. Patients either underwent surgical resection (stage I/II), stereotactic radiotherapy (stage I), combined chemo-radiotherapy (stage III) or chemotherapy alone (stage IV). Stage IV patients with epidermal growth factor receptor (EGFR) mutations were treated with tyrosine kinase inhibitors. The available tissue blocks of formalin-fixed paraffin embedded tumor were collected. Peripheral blood mononuclear cells (PBMCs) were isolated by Ficoll density centrifugation and subsequently cryopreserved in liquid nitrogen [14, 15]. In case of surgical resection, fresh tissue from the primary tumor and its draining lymph node were obtained.

### XAGE-1b immunohistochemistry (IHC)

Tumor blocks were cut in 4-µm sections and deparaffinized in xylene. Endogenous peroxidase activity was blocked by incubation in 0.3 % hydrogen peroxide/methanol for 10 min at room temperature (RT). Antigen retrieval was performed by heating the samples to 97 °C for 30 min in citrate buffer (pH 6.0, DAKO, Glostrup, Denmark), cooled on ice and incubated at RT for 1 h with 2 µg/ml XAGE-1 mouse monoclonal antibody LX199#5 (kindly provided by the Ludwig Institute for Cancer Research, LICR) in phosphate-buffered saline (PBS, Fresenius Kabi Bad Homburg, Germany) with 1 % bovine serum albumin (BSA). After washing, the slides were incubated with horseradish peroxidase-conjugated anti-mouse IgG (DAKO envision) for 30 min at RT. NovaRed (Vector, Burlingame, USA) was applied as a chromagen, and sections were counterstained with Mayer’s hematoxylin (Klinipath). All slides were mounted with Pertex mounting medium (HistoLab, Sweden). All washing steps were done with PBS.

XAGE-1b expression was scored according to a previously described method [11] as negative (<5 % cancer cells positive), focal (5–10 % positive), intermediate (11–50 % positive) or diffuse (>50 % positive). From each slide, 10 random tumor fields (magnification 20×, approximately the size of a biopsy) were scored for XAGE-1b expression. When available, tissue blocks from multiple tumor sections were assessed.

### XAGE-1b protein and overlapping peptides

*E. coli*-produced recombinant XAGE-1b protein (81 amino acids) was obtained using a XAGE-1 plasmid DNA, kindly provided by the LICR. Five synthetic overlapping peptides covering the entire sequence of the XAGE-1b protein were synthesized at the LUMC by solid-phase strategies on an automated peptide synthesizer (Abimed AMS 422, Germany) using Fmoc chemistry. Peptides were analyzed by reverse-phase HPLC, dissolved in DMSO at 50 mg/ml, aliquoted and stored at −80 °C until use. The amino acid sequences of the five peptides are:

- p1, amino acid 1-32, MESPKKKNQQLKVGILHLGSRQKKIRIQLRSQ;
- p2 amino acid 18-42, LGSRQKKIRIQL-RSQCATWKVICKS;
- p3, amino acid 34-59, ATWKVICKSCISQTPGINLDLGSGVK;
- p4, amino acid 37-68, KVICKSCISQTPGINLDLGSGVKVKIIPKEEH;
- p5, amino acid 55-81, GSGVKVKIIPKEEHCKMPE-AGEEQPQV.

Working solutions were prepared at a concentration of 2.5 mg/ml and stored at −20 °C.

### Detection of XAGE-1b-specific IgG antibodies

Serum samples were analyzed for XAGE-1b peptide-specific immunoglobulin G (IgG) by ELISA. Serum samples with known high IgG titers for XAGE-1b (KLU 187, kindly provided by Dr. E. Nakayama, Okayama University, Japan) were used to develop the ELISA. One serum sample (X-14) with proven high XAGE-1b IgG titers was included in each experiment as a positive control. All washing steps were done with PBS. A 96-well EIA/RIA plate (Costar 3590) was coated overnight at 4 °C with 50 μl of the individual XAGE-1b peptides and a mix of all peptides (5 μg/ml of each peptide diluted in 0.1 M carbonate/bicarbonate coating buffer; Merck, Darmstadt, Germany). The next day, non-specific binding sites of the plate were blocked with 5 % FCS/PBS (100 μl/well, fetal calf serum, PAA laboratories, Austria) for 1 h at RT. Subsequently, serially diluted serum samples (1:100, 1:500, 1:1000, 1:2000, 1:4000 in blocking buffer) were added in triplicate wells (50 μl/well) and incubated at RT for 2 h. Next, goat antihuman IgG-horseradish peroxidase (HRP, Southern Biotechnology, Birmingham, AL) was added (diluted 1:3000 in blocking buffer) and incubated for 1 h at RT. Finally, tetramethylbenzidine liquid substrate (Sigma Aldrich, 50 μl/well) was added for the colorimetric enzymatic reaction, which was stopped by adding 50 μl/well of 2 M H_2_SO_4_ (Merck), and the plate was read in an ELISA reader at 450 nm. The average OD value of the triplicate uncoated wells (background value) was calculated and an outlier per triplicate was discarded when the value exceeded the average plus 2× standard deviation (SD). A positive XAGE-1b peptide-specific IgG response was defined as an average of the triplicate wells which was at least twofold above background value.

### Cell culture

The tumor-infiltrating lymphocytes (TILs) culture method has been published previously [14, 15]. Briefly, TILs were isolated by mincing fresh tumor tissue into pieces followed by a 2- to 3-week homeostatic in vitro culture in Iscove’s modified Dulbecco’s medium (IMDM) supplemented with 10 % human AB (hAB) serum (PAA laboratories) and a mix of homeostatic cytokines: 10 % T cell growth factor (TCGF, Zeptometrix, USA), 5 ng/ml of interleukin-15 (IL-15; Peprotech) and (only on day 1) 5 ng/ml of IL-7 (Peprotech). Lymph node (LN) mononuclear cells were isolated from tumor-draining lymph nodes and cultured for a week in medium alone (LN neg) or supplemented with a mix of 5 XAGE-1b overlapping peptides (LN XAGE, 2.5 µg/ml/peptide), after which the T cells were expanded with recombinant human IL-2 (150 IU/ml, refreshed 3 times per week) for 2–3 weeks. After harvesting, the T cells were evaluated for presence of XAGE-1b-specific CD4+ CD25+ Foxp3^high^ T cells, XAGE-1b-specific proliferation and cytokine production.

### Detection of XAGE-1b-specific CD4CD25+ Foxp3^high^ T cells

PBMCs, TILs and LN cells were stained for CD4, CD8, Foxp3 and CD25 as previously reported [16, 17].

### Analysis of XAGE-1b-specific T cells by proliferation assay and cytokine profile

T cells (50,000/well) were stimulated with autologous monocytes pulsed with XAGE-1b peptides and/or protein (5 μg/ml) in triplicate wells in a three-day proliferation assay [14] with phytohaemagglutinin (PHA) (Remel, Germany) used as positive control. Supernatants collected after 2 days were analyzed for Th1/Th2 cytokines (IFNγ, TNF-α, IL-10, IL-5, IL-4, IL-2) by cytometric bead array (CBA, BD Biosciences). Proliferation was measured by ^3^H-thymidine incorporation (0.5 μCi/well) during the last 18 h of the assay [18]. The stimulation index (SI) was calculated by taking mean counts of stimulated wells divided by mean counts of the medium control wells. A XAGE-1b-specific T cell response was defined by either a SI index of >3 or by XAGE-1b-specific cytokine production, which was defined as a cytokine concentration above the cutoff value (20 pg/ml, except for IFNγ for which the cutoff was 100 pg/ml) and more than twice the concentration of medium control [19].

### ELISPOT

Details of the four-day IFNγ ELISPOT assay have been reported previously [17]. Spots were counted with a fully automated computer-assisted video imaging analysis system (BioSys 5000). Specific spots were calculated by subtracting the mean number of spots in quadruplicate wells + 2 × SD of the medium only control from the mean number of spots in test wells. Antigen-specific T cell frequencies were considered to be increased compared to medium control when specific T cell frequencies were ≥1/10,000 [17].

### Analysis of XAGE-1b-specific T cells by multiparameter flow cytometry

T cells were stimulated overnight with autologous monocytes pulsed with individual XAGE-1b peptides (5 μg/ml), a XAGE-1b peptide mix (5 μg/peptide/ml) and XAGE-1b protein (10 μg/ml). E7 protein of the human papillomavirus type 16 (HPV16 E7) was used as negative control. The percentage and polarization of XAGE-1b-specific T cells was measured by simultaneous staining for T cell markers (CD3, CD4 and CD8), T cell activation markers (CD137 and CD154) and cytokines (IFNγ, IL-2) according to standard operating procedures (SOPs) [14, 16, 20]. XAGE-1b-specific T cells were detected when the percentage of XAGE-1b-specific CD4+ CD154+ CD137+, CD8+ CD137+ or cytokine-producing T cells was at least twice the percentage detected in the medium control. The responding cells were visible as a clearly distinguishable population in the flow cytometry contour plot. An example of a gating strategy is provided in Supplementary Figure 1.

### Isolation of XAGE-1b-specific T cell clones

T cell clones were isolated from PBMCs using limiting dilution as described earlier [15]. Specificity of T cell clones for XAGE-1b was tested by proliferation assay on peptide or protein-loaded irradiated autologous Epstein–Barr virus (EBV)-transformed B cell lines (B-LCL) or autologous monocytes. Furthermore, clones were tested by flow cytometry for phenotype (CD4/CD8) and T cell receptor Vβ (TCRVβ) expression using a TCRVβ kit (Beckman Coulter) comprising eight sets of antibodies, each consisting of three differently labeled antibodies specific for three different TCRVβ families, ultimately covering about 70 % of the normal human TCRVβ repertoire. A TCRVβ was considered dominant (>10 %), subdominant (3–10 %) or minor (<3 %) based on the percentage of T cells using the same TCRVβ [14, 21].

## Results

An overview of patient characteristics is presented in Table 1. The mean age was 65.9 years (range 45–82 years), and the male/female ratio was 20/20.

**Table 1:** Overview of patient characteristics, XAGE-1b immunohistochemistry and IgG response

ID	Stage	Treatment^1^	Tumor material	XAGE-1b IHC^2^	Number of + fields^3^	Number of + sections^4^	Metastasis	XAGE-1b IgG^5^
X-1	I	RT	Biopsy	Negative				–
X-2	IV	CT	Biopsy	Negative				–
X-3	IIIa	CT-RT	Biopsy	Negative				–
X-4	IIIb	CT	Biopsy					+
X-5	I/II	Surgery	Resection	Focal	2	1 of 3		–
X-6	IV	CT	Biopsy	Negative				–
X-7	I/II	Surgery	Resection	Focal	4	1 of 1		–
X-8	IIIa	CT-RT	Biopsy	Negative				–
X-9	I/II	Surgery	Resection	Intermediate	10	1 of 1		–
X-10	IIIb	CT-RT	Biopsy	Negative				–
X-11	IV	CT	Metastasis				Negative	–
X-12	I/II	Deceased	Biopsy	Focal				–
X-13	I/II	Surgery	Resection	Negative	0			–
X-14	I/II	Surgery	Resection	Diffuse	10	2 of 3	Diffuse	+
X-15	I/II	Surgery	Resection	Focal	4	1 of 1		–
X-16	IIIa	CT-RT	Metastasis				Negative	–
X-17	I/II	Surgery	Resection/Metastasis	Diffuse	6	2 of 2	Negative	–
X-18	I/II	Surgery	Resection/Metastasis	Negative	0	0 of 2	Negative	–
X-19	I/II	Surgery	Resection	Focal	3	1 of 3		–
X-20	I/II	Surgery	Resection	Negative	0	0 of 3		–
X-21	I/II	Surgery	Resection	Intermediate	4	2 of 2		–
X-22	I/II	Surgery	Resection	Intermediate	3	2 of 2		–
X-23	I/II	Surgery	Resection	Negative	0	0 of 2		–
X-24	I/II	Surgery	Resection	Intermediate	6	2 of 2		–
X-25	I/II	Surgery	Resection	Negative	0	0 of 2		–
X-26	I/II	Surgery	Resection	Intermediate	4	1 of 2		
X-27	I/II	Surgery	Resection	Negative	0	0 of 6		+
X-28	I/II	Surgery	Resection	Negative	0	0 of 4		–
X-29	I/II	Surgery	Resection	Negative	0	0 of 2		–
X-30	I/II	Surgery	Resection	Intermediate	3	1 of 3		–
X-31	I/II	Surgery	Resection	Negative	0	0 of 1		–
X-32	I/II	Surgery	Resection	Negative	0	0 of 3		–
X-33	I/II	Surgery	Resection	Negative	0	0 of 5		–
X-34	IV	TKI	Metastasis				Diffuse	–
X-35	I/II	Surgery	Resection	Diffuse	6	1 of 1		–
X-36	I/II	Surgery	Resection	Diffuse	7	4 of 4		–
X-37	I/II	Surgery	Resection	Diffuse	6	3 of 3		–
X-38	I/II	Surgery/CT	Resection/Metastasis	Negative	0	0 of 2	Negative	–
X-39	IV	CT	Biopsy	Negative				–
X-40	I/II	Surgery	Resection	Negative	0	0 of 4		–

^1^ *CT* chemotherapy, *CT*-*RT* combined chemo-radiotherapy, *RT* radiotherapy, *TKI* tyrosine kinase inhibitors

^2^XAGE-1b immunohistochemistry (IHC) scored as negative (<5 % positive), focal (5–10 %), intermediate (11–50 %) and diffuse (>50 %). *NT not tested*

^3^10 random tumor fields (magnification 20×) from each slide were scored. The number (Nr.) of positively scored fields are given. *NT not tested*

^4^When available, XAGE-1b overexpression was assessed in multiple tumor sections

^5^XAGE-1b-specific IgG antibody response is shown

### XAGE-1b expression in lung adenocarcinoma

In our patient cohort, the whole primary tumor was available for XAGE-1b staining from 28 patients who underwent surgical resection (stage I/II). A biopsy of the primary tumor was available in nine cases (two stage I/II patients and seven stage III/IV patients). In three cases, XAGE-1b expression was assessed on metastasized tissue only. In one patient (X-4), the biopsy of the primary tumor could not be retrieved, and hence, XAGE-1b status was not assessed. Overall, in 17 of 39 evaluable cases (43.6 %), XAGE-1b expression was observed: focal (*n* = 5), intermediate (*n* = 6) and diffuse (*n* = 6). Positive staining was found in the primary tumor (*n* = 15), metastatic tissue (*n* = 1) or both (*n* = 1) (Table 1). The XAGE-1b staining pattern was always nuclear, and occasionally, also cytoplasmic staining was observed. An example of XAGE-1b staining is presented in Supplementary Figure 2. While 54 % (15 of 28) of the resected tumor specimens stained positive for XAGE-1b, this was only the case for one out of eight biopsies evaluated. To assess whether the XAGE-1b status in biopsies truly reflects the XAGE-1b status of the primary tumor, 10 random tumor fields of the resected tumor specimens (*n* = 28; magnification 20×, approximately the size of a biopsy) were scored for XAGE-1b overexpression. While none of the 13 previously scored negative tumors showed XAGE-1b staining in the 10 random fields, the 15 XAGE-1b-positive resected tumors displayed XAGE-1b staining in (on average) 5.2 out of 10 fields (range 2–10). In addition, multiple tissue blocks of the same tumor (average 3.0 blocks, range 2–6) were studied for 22 of 28 operated patients. In 17 of 22 cases, staining score (positive or negative) was identical in all blocks from the same case (Table 1). Our data demonstrate that XAGE-1b overexpression is found in about 40 % of all tumors; however, positive tumors do not show overexpression in all randomly selected tumor fields.

### XAGE-1b-specific T cells are present in the primary lung tumor and its draining lymph nodes

Fresh samples of tumor and lymph node tissue were collected from 24 of the 28 stage I/II adenocarcinoma patients. In 20 cases, we successfully expanded TILs. Tumor-draining lymph node (LN) mononuclear cells were expanded in vitro in the presence (LN XAGE) or absence (LN neg) of exogenous XAGE-1b peptides. The phenotype (CD4, CD8, Foxp3) and activation status (i.e., CD25 expression) of these T cells are presented in Supplementary Figure 3.

**Fig. 1:**
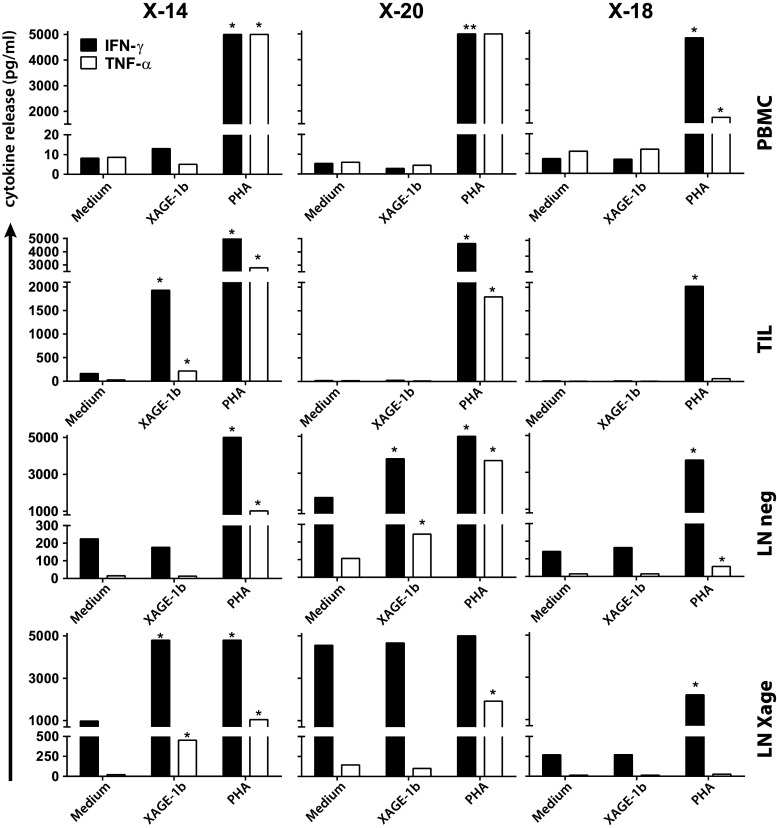
Local XAGE-1b-mediated immunity, Th1 response. Day 2 supernatants from PBMCs, TILs and LN cells, co-cultured with XAGE-1b pulsed monocytes, were analyzed for Th1 (IFNγ and TNF-α) and Th 2 (IL-5 and IL-10) cytokine release. A positive response (indicated with *asterisks*) was defined by a cytokine concentration above the cutoff value (20 pg/ml, except for IFNγ, 100 pg/ml) and more than twice the concentration of medium control. Depicted here are the Th1 cytokines. XAGE-1b-specific Th1 cytokine release was observed in TILs (X-14) and LN cells (X-14, X-20). Included are the results from one negative (X-18) patient. PHA was used as positive control

Next, the presence of local (TILs/LN neg/LN XAGE) as well as circulating XAGE-1b-specific T cells (PBMCs) was investigated by analysis of XAGE-1b-specific proliferation and cytokine secretion in these 20 patients. In one patient (X-14) with diffuse (>50 %) XAGE-1b staining in the primary tumor, a T helper 1 (Th1) response to XAGE-1b was detected (Fig. 1). XAGE-1b-specific secretion of IFNγ and TNFα was detected in both TILs and LN XAGE cells after co-culture with autologous monocytes pulsed with a mix of XAGE-1b overlapping peptides. In addition, the T helper type 2 (Th2) cytokines IL-5 and IL-10 were secreted (Supplementary Figure 4). Despite the XAGE-1b-specific high production of cytokines, the proliferative response was low in TILs (SI index 0.7) and LN XAGE (SI 2.1), indicating that these T cells had poor proliferative capacity after their initial expansion (data not shown).

Another patient (X-20), without XAGE-1b expression in the primary tumor, showed XAGE-1b-specific T cell reactivity in its draining LN. XAGE-1b-specific IFNγ and TNFα production was detected in the LN cells only expanded with IL-2 (LN neg) (Fig. 1), and these T cells also produced IL-5 but not IL-10 (Supplementary Figure 4). A XAGE-1b-specific response in the LN cells cultured with XAGE-1b peptides (LN XAGE) was not demonstrated potentially due to the high background cytokine production in these activated T cells after culture (Fig. 1). Again, despite high cytokine release in the LN neg cells, the XAGE-1b-specific proliferative response was low (SI 1.2, data not shown).

Overall, we found local XAGE-1b-mediated Th1/Th2 cell immunity in two of 20 patients tested indicating that XAGE-1b acts as a genuine tumor antigen.

### Identification of XAGE-1b B cell epitopes

**Fig. 2:**
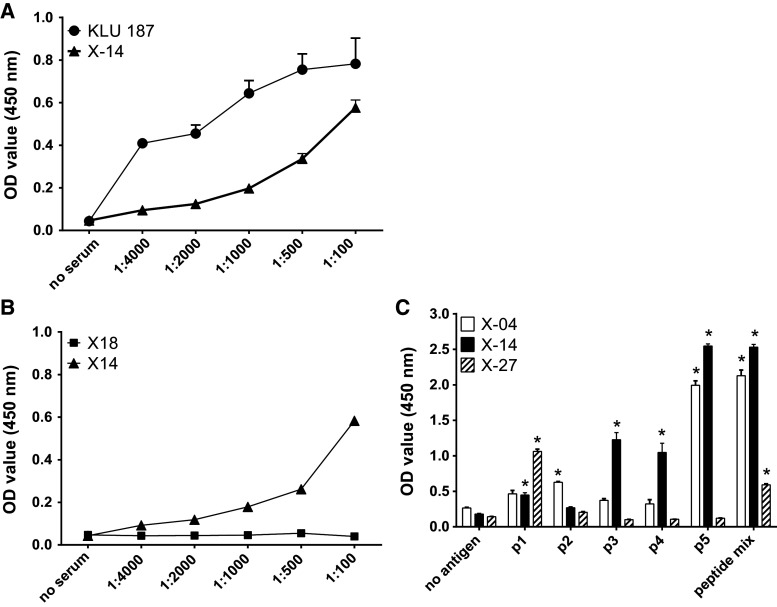
XAGE-1b-specific IgG antibodies in patient sera. **a** Example of a XAGE-1b-specific IgG response (X-14) to XAGE-1b peptide mix. KLU 187 represents a serum sample previously shown to have high IgG antibody titers [13] and was used as positive control to set up the ELISA. Serial dilutions are shown. **b** Example of a patient (X-18) with no XAGE-1b IgG response to XAGE-1b peptide mix. X-14 was used as positive control. **c** IgG response (serum 1:100 diluted) to individual XAGE-1b peptides in 3 of the 40 tested patients shows broad recognition. A positive response was defined as at least a twofold increase compared to background (no antigen) and is indicated with *asterisks*

The ELISA for measuring XAGE-1b-specific IgG antibodies was developed with a high IgG titer serum sample (KLU 187) [13] as positive control. A relatively strong XAGE-1b-specific IgG response was detected in patient X-14 (Fig. 2). In total, 3 out of 40 evaluated patients (7.5 %) displayed a XAGE-1b-specific IgG response when tested against a mix of the 5 XAGE-1b overlapping peptides. These three serum samples were also tested against the individual XAGE-1b peptides (Fig. 2c). Although peptide p5 showed the strongest response, the sera showed a response to multiple epitopes in XAGE-1b.

Of these three patients, one patient (X-14) expressed XAGE-1b in the tumor and mounted a local XAGE-1b-specific T cell response. Surprisingly, the second patient (X-27) did not show XAGE-1b expression in the tumor, despite the analysis of six separate tumor sections. For the last patient (X-4), the XAGE-1b tumor status could not be assessed due to unavailability of the tumor sample.

### XAGE-1b-specific T cells are present in peripheral blood both direct ex vivo and after in vitro expansion

Since the presence of IgG antibodies indicate an underlying T cell response [13], the three patients (X-4, X-14, X-27) with XAGE-1b-specific IgG were analyzed for circulating XAGE-1b-specific T cells. First, PBMCs were stimulated with the pool of XAGE-1b peptides or medium only and examined in a direct ex vivo IFNγ ELISPOT. In one patient (X-4), a strong XAGE-1b-specific IFNγ response was demonstrated (Supplementary Figure 5) indicative for the presence of circulating XAGE-1b-specific T cells. PBMCs of the other patients (X-14, X-27) did not show a direct ex vivo XAGE-1b-specific T cell response (data not shown).

**Fig. 3:**
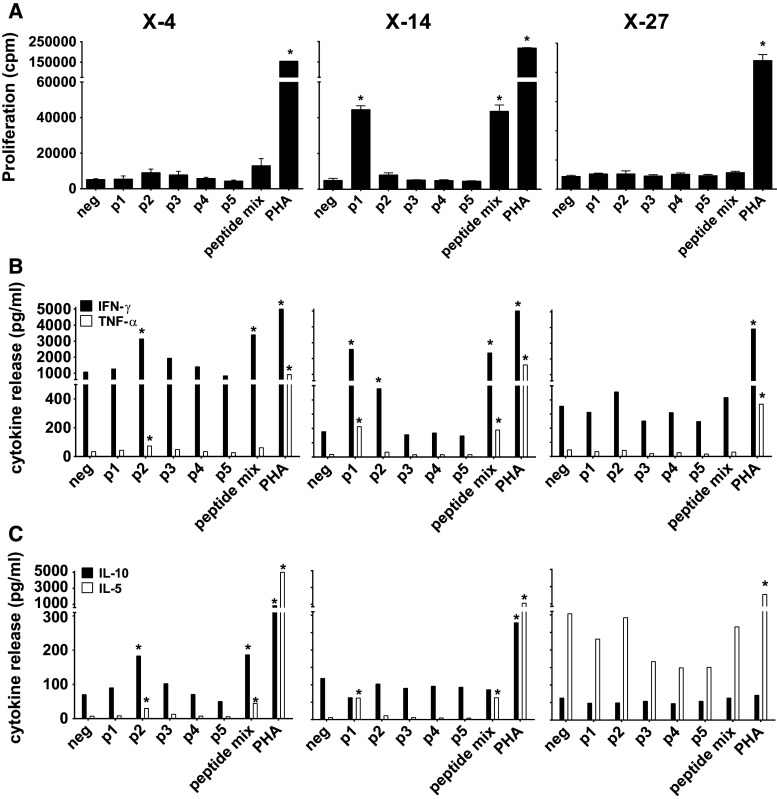
Circulating XAGE-1b-specific T cells: proliferation and release of type I/II cytokines. **a** PBMC samples from three patients (X-4, X-14, X-27) with XAGE-1b-specific IgG antibodies were stimulated with XAGE-1b overlapping peptides in a 10-day culture and subsequently tested for XAGE-1b-specific proliferation. A positive response (indicated with asterisk) was defined as a SI index of ≥3. Patient X-14 showed a response specific for peptide p1 and XAGE-1b peptide mix, X-4 showed a modest proliferative response to the XAGE-1b peptide mix, whereas X-27 showed no proliferative response at all. **b** Release of Th1 cytokines (IFNγ, TNFα) was observed in two patients (X-4, X14) specific for peptide p1, p2 and peptide mix. **c** Release of Th2 cytokines (IL-10, IL-5) was observed in two patients (X-4, X14) and was specific for peptide p1, p2 and peptide mix. The third patient (X-27) showed no detectable response (**b**, **c**)

Subsequently, PBMC samples of these three patients were stimulated with the mix of XAGE-1b-overlapping peptides in vitro for 10 days and then tested for XAGE-1b-specific proliferation and Th1/Th2 cytokine production (Fig. 3). Patient X-4 showed a Th1 response to XAGE-1b reflected by the production of high amounts of IFNγ and low levels of IL-5 and IL-10 to peptide p2 and to the mix of five peptides. Similar to our previous observation, the proliferative response of the T cells was modest at best to the peptide mix (SI 2.5) and undetectable to individual peptides. The PBMC culture of patient X-14 produced large amounts of IFNγ when stimulated with peptide p1, p2 or the peptide mix as well as low amounts of TNFα and IL-5 upon stimulation with p1 and the peptide mix. The PBMCs of this patient proliferated when stimulated with p1 (SI 8.5) and peptide mix (SI 8.4) but not when stimulated with p2.

The third patient (X-27) neither showed cytokine production nor a proliferative response specific for XAGE-1b (Fig. 3). However, we observed an unusually high concentration of CD14+ myeloid cells in the peripheral blood of this patient. Myeloid-derived suppressor cells have been shown to be elevated and to have a suppressive effect on T cells in NSCLC [22, 23]. Therefore, the CD14+ cells were removed from the PBMCs by magnetic-activated cell sorting (MACS), and the remaining cells were stimulated with the XAGE-1b peptide mix. As a result, the 10-day cultured PBMCs produced IFNγ, TNFα and IL-5 and proliferated upon stimulation with p2 (SI 3.9) and the XAGE-1b peptide mix (SI 4.1) (Supplementary Figure 6). Thus, XAGE-1b-specific T cells are present in the peripheral blood of XAGE-1b IgG-positive patients, but their reactivity can be obscured by a CD14+ myeloid population.

### Type and specificity of the XAGE-1b-specific T cell response

**Fig. 4:**
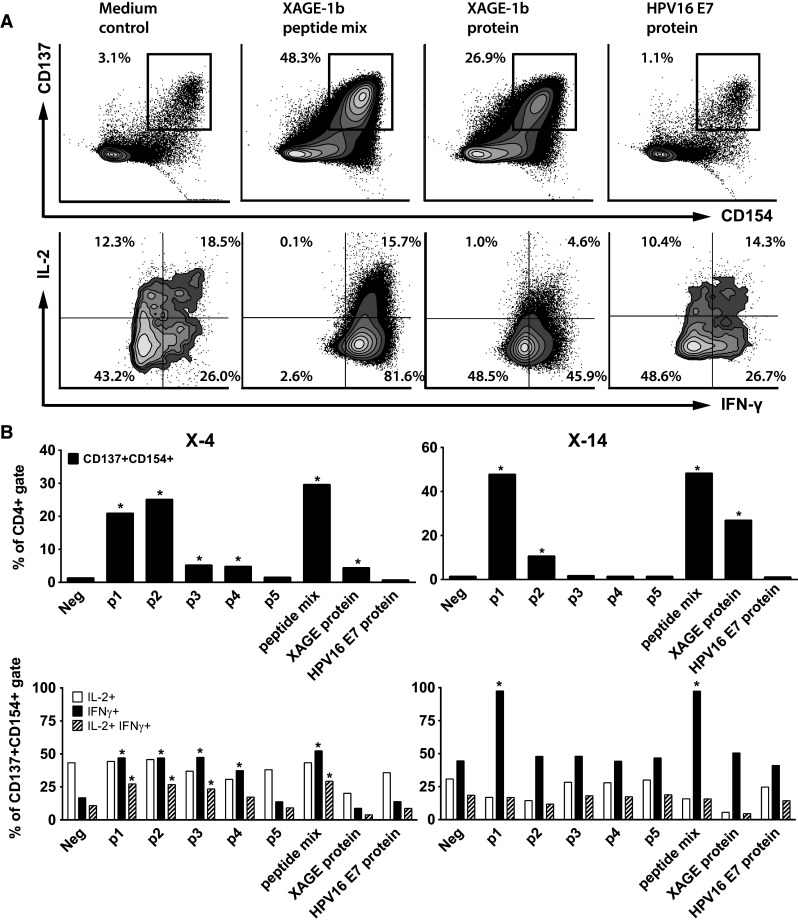
Type and specificity of XAGE-1b-specific CD4 T cell response. **a** The CD4+ T cells in the 8-week cultured PBMCs of patient X-14 were gated (see Supplementary Figure 1). Subsequently, CD4+ double-positive (CD137+ CD154+) T cells were gated. Finally, the intracellular IFNγ/IL-2 production within this population was plotted. Upon stimulation with XAGE-1b peptide mix and XAGE-1b, a specific upregulation of the T cell activation markers CD137/CD154 and the intracellular expression of IFNγ and IL-2 are demonstrated as compared to medium control and the negative control protein (HPV16 E7). **b** The specificity of CD4+ CD137+ CD154+ T cells for individual peptides and XAGE-1b protein and type I intracellular cytokine profile are shown for two patients (X-4 and X-14). *Asterisks* indicate positive responses (at least twice the percentage detected in the medium control). For patient X-4, a CD4+ T cell response was observed specific for peptide p1, p2 and the peptide mix, as well as a weak response to p3, p4 and p5. Patient X-14 displayed a strong CD4+ T cell response when stimulated with p1, peptide mix and XAGE-1b protein as well as a moderate response to p2

To type and enumerate XAGE-1b-specific T cells in 8-week cultured PBMCs, we assessed the frequency of CD4+ and CD8+ T cells with increased expression of the T cell activation markers CD137 and CD154 after stimulation with antigen-pulsed autologous monocytes (Fig. 4a). CD4+ T cells of patient X-4 showed a strong response toward stimulation with p1, p2 and the peptide mix. A weak response was observed to p3, p4 and XAGE-1b protein. The type I cytokines IFNγ and/or IL-2 were produced by the double-positive (CD137+ CD154+) T cells (Fig. 4b) upon stimulation with p1, p2, p3, p4 and the peptide mix. Unfortunately, the number of expanded CD8+ T cells in this culture was too low to thoroughly assess XAGE-1b-specific CD8+ T cell reactivity.

The cultured PBMCs of patient X-14 showed a strong CD4+ T cell mediated response when stimulated with p1, peptide mix and XAGE-1b protein as well as a moderate response to p2, corroborating the earlier data (Fig. 4b). The cytokine profile of double-positive T cells mainly showed high IFNγ production specific for p1 and the peptide mix. Notably, the CD8+ T cells within this culture reacted to p1, the peptide mix and XAGE-1b protein (Supplementary Figure 7). Surprisingly, the peptide- and protein-specific IFNγ production in these CD8 T cells is not accompanied by an increased expression of the activation marker CD137 (Supplementary Figure 8).

Subsequently, T cell clones were isolated from the bulk-cultured PBMCs from patients X-4 and X-14. During the cloning procedure, the expanded PBMCs were kept in culture, and subsequently, the established clones and bulk-cultured PBMCs were characterized with respect to TCR-Vβ usage and antigen-specific proliferation. Analysis of TCR-Vβ usage showed the presence of at least 11 different TCR-Vβ families of CD4+ T cells in the bulk-cultured PBMCs of X-4 (Supplementary Figure 9A). Upon stimulation with peptide p1, p2, the peptide mix and XAGE-1b protein, the bulk-cultured PBMCs of this patient showed a proliferative response (Supplementary Figure 9B). Only one XAGE-1b-specific CD4+ T cell clone could be established (Supplementary Figure 9C). This clone (X-4.6) responded to p1, p2 and the peptide mix. The TCR-Vβ analysis did not lead to the identification of a specific TCR-Vβ family usage (data not shown), indicating that the TCR-Vβ used was outside the range of families covered by the eight sets of antibodies.

**Fig. 5:**
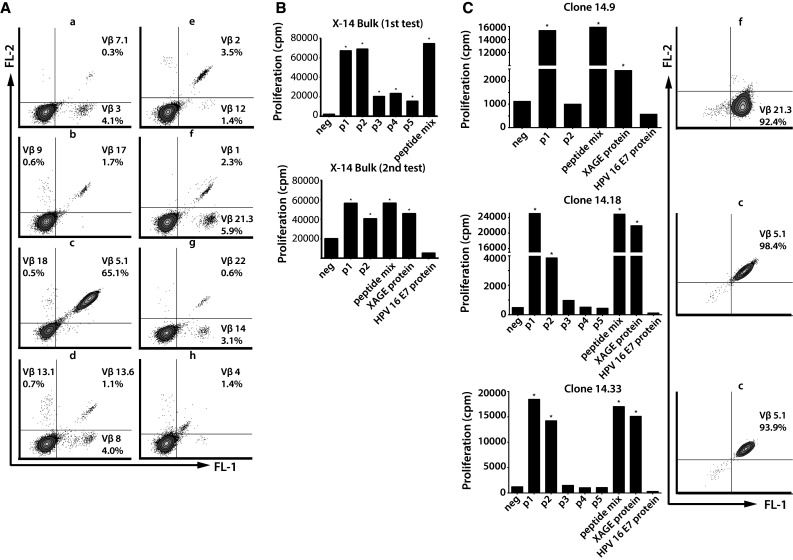
TCR-Vβ expression and XAGE-1b-specific proliferation of bulk-cultured PBMCs of patient X-14. **a** Sixteen different TCR-Vβ families were discovered in the bulk-cultured PBMCs of patient X-14. **b** XAGE-1b-specific proliferation (conducted in two separate assays) demonstrated a broad response to all 5 overlapping XAGE-1b peptides, the peptide mix and to XAGE-1b protein. **c** In total, 10 clones were isolated from the expanded PBMCs; XAGE-1b-specific proliferation and TCR-Vβ usage are shown for three clones with different patterns of antigen recognition (p1, p2, the peptide mix and XAGE-1b protein) and TCR-Vβ expression (Vβ 21.3 and 5.1)

The CD4+ T cells in the bulk-cultured PBMCs of patient X-14 displayed the use of at least 16 different TCR-Vβ-families (Fig. 5a), of which one was considered dominant (Vβ5.1) and five were considered subdominant (Vβ2/3/8/14/21.3). The bulk-cultured PBMCs showed a broad response to all five overlapping XAGE-1b peptides, the peptide mix and to XAGE-1b protein (Fig. 5b). A total of 10 XAGE-1b-specific CD4+ T cell clones were obtained of which eight were analyzed for TCR-Vβ usage. Staining for the dominant TCR-Vβ 5.1 was demonstrated for four clones, whereas the subdominant TCR-Vβ 21.3 was expressed by one clone (Fig. 5c). The established clones showed XAGE-1b-specific proliferation when stimulated with p1, p2, the peptide mix and XAGE-1b protein, indicating that CD4+ T cell clones isolated from this culture recognized their naturally processed cognate antigen. Flow cytometric analysis of intracellular Foxp3 expression by the isolated clones did not reveal the presence of this transcription factor (data not shown).

Taken together, we conclude that T cell reactivity to XAGE-1b involves polyclonal CD4+ and CD8+ T cell populations with the capacity to recognize the naturally processed antigen and reactive to peptide epitopes different from those recognized by the IgG antibodies.

## Discussion

This is the first study on the immunogenicity of XAGE-1b in a Caucasian study cohort of 40 pulmonary adenocarcinoma patients. We found XAGE-1b protein expression to be present in 43.6 % of cases, which is within the frequency range (33–53 %) reported for Asian patients [10, 11, 24]. XAGE-1b was expressed in both primary and metastatic tumor specimens. However, XAGE-1b-positive tumors do not show protein overexpression in all tumor samples nor in all fields covering an area that approximates the size of a bronchial biopsy. Hence, the frequency of XAGE-1b-overexpressing tumors can be underestimated when only assessing a single section of the tumor and most likely when a biopsy is analyzed. Based on our findings, showing that sometimes only 2 out of 10 randomly analyzed tumor fields stain positive for XAGE-1b, its status is probably best assessed by the analysis of at least two sections of the primary tumor or five biopsies. Moreover, our study reveals for the first time the presence of XAGE-1b-specific T cells in the primary lung tumor and the tumor-draining lymph nodes from 2 out of 20 evaluated patients. The XAGE-1b-reactive T cells displayed both a Th1 (IFNγ/TNFα) and Th2 (IL-5/IL-10) cytokine polarization. These results indicate that XAGE-1b-specific T cells can contribute to the anti-tumor response, but the low response rate among patients also implies that the spontaneous activation of T cells to XAGE-1b is limited.

In our cohort, we observed XAGE-1b-specific IgG antibodies in 3 of 40 patients (7.5 %). Two previous studies with Asian adenocarcinoma patients also assessed anti-XAGE-1b antibody frequency. The first study [11] found a XAGE-1b IgG frequency of 8.9 % in a similar (mainly stage I/II) patient group. The second study [13] found a higher frequency (19 %) of XAGE-1b IgG responses, but this was in stage IIIb/IV patients. Possibly, XAGE-1b-specific antibodies are more prevalent in patients with a more advanced tumor stage, as also observed for antibodies against p53, NY-ESO-1 and survivin [25].

The presence of XAGE-1b-specific IgG antibodies was accompanied by an antigen-specific T cell response in the peripheral blood of all three antibody-positive patients. In one patient (X-4), an ex vivo IFNγ response to XAGE-1b peptide mix was found by ELISPOT assay, whereas in two patients (X-4 and X-14), Th1 and Th2 cytokine responses were found after one round of in vitro stimulation of PBMCs with XAGE-1b overlapping peptides. In-depth analysis revealed that the XAGE-1b-specific T cell population comprised both CD4 and CD8 T cells producing both Th1 and Th2 cytokines. The T cells reacted mainly to the overlapping peptides p1 and p2. Recently, this N-terminal part of XAGE-1b was shown to comprise a CD4+ T cell epitope (aa 18–31) and an overlapping CD8+ T cell epitope (aa 21–29) [13]. Our study confirms this part of XAGE-1b as a T cell epitope-containing domain. Interestingly, while the T cell response was predominantly directed against sequences present in p1 and p2, the dominant B cell response was targeted to p5 of XAGE-1b within the small group of patients positive in this study.

In one patient (X-27, Supplementary Figure 6), an underlying XAGE-1b-specific T cell response was only detected after removal of a myeloid CD14+ population from the PBMC sample. Immature myeloid cell populations have been shown to inhibit T cell activation [26, 27], and their suppressive activity has been specifically reported for NSCLC [22, 23, 28]. Therefore, removal of the potentially suppressive myeloid cell populations from the peripheral blood of adenocarcinoma patients before analysis of T cell responses might reveal a higher percentage of XAGE-1b-specific T cell responders among Caucasian patients. In addition, based on the current success with co-inhibitory receptor blocking in patients with NSCLC [7], one could consider to test the response in assays where known co-inhibitory receptors are blocked.

In conclusion, our study demonstrates that XAGE-1b acts as a genuine tumor antigen eliciting integrated systemic and/or tumor-infiltrating antigen-specific humoral and cellular immune responses in Caucasian patients with pulmonary adenocarcinoma. As such, this tumor antigen forms an attractive target for active immunotherapy in lung cancer using XAGE-1b-based therapeutic vaccines. Based on the presence of these integrated XAGE-1b-specific responses in non-vaccinated patients, it is not to be expected that the induction of such a response by XAGE-1b vaccination will result in safety problems. Based on these results, we have recently started a phase 1 clinical trial with XAGE-1b synthetic long peptides.

### Electronic supplementary material

Below is the link to the electronic supplementary material.

Supplementary material 1 (PDF 1587 kb)

## Abbreviations

APC: Antigen-presenting cell
B-LCL: EBV transformed B cell lines
CBA: Cytometric bead array
CT: Computed tomography
ELISA: Enzyme-linked immunosorbent assay
ELISPOT: Enzyme-linked immunosorbent spot
FCS: Fetal calf serum
Foxp3: Forkhead box P3
IHC: Immunohistochemistry
LICR: Ludwig Institute for Cancer Research
LN: Lymph node
LUMC: Leiden University Medical Center
NSCLC: Non-small cell lung cancer
PBMCs: Peripheral blood mononuclear cells
PHA: Phytohaemagglutinin
RT: Room temperature
TCGF: T cell growth factor
TCR: T cell receptor
TKI: Tyrosine kinase inhibitors
TILs: Tumor-infiltrating lymphocytes
