# Supplementary material for: Local and systemic XAGE-1b-specific immunity in patients with lung adenocarcinoma
Source: Cancer Immunol Immunother. 2015 May 30;64(9):1109–21. doi: 10.1007/s00262-015-1716-2 (PMC4540777; doi:10.1007/s00262-015-1716-2)
Supplement: Supplementary file 1 — Supplementary material 1 (PDF 1587 kb) [file 262_2015_1716_MOESM1_ESM.pdf]

Supplementary Figure 1. Example of gating strategy

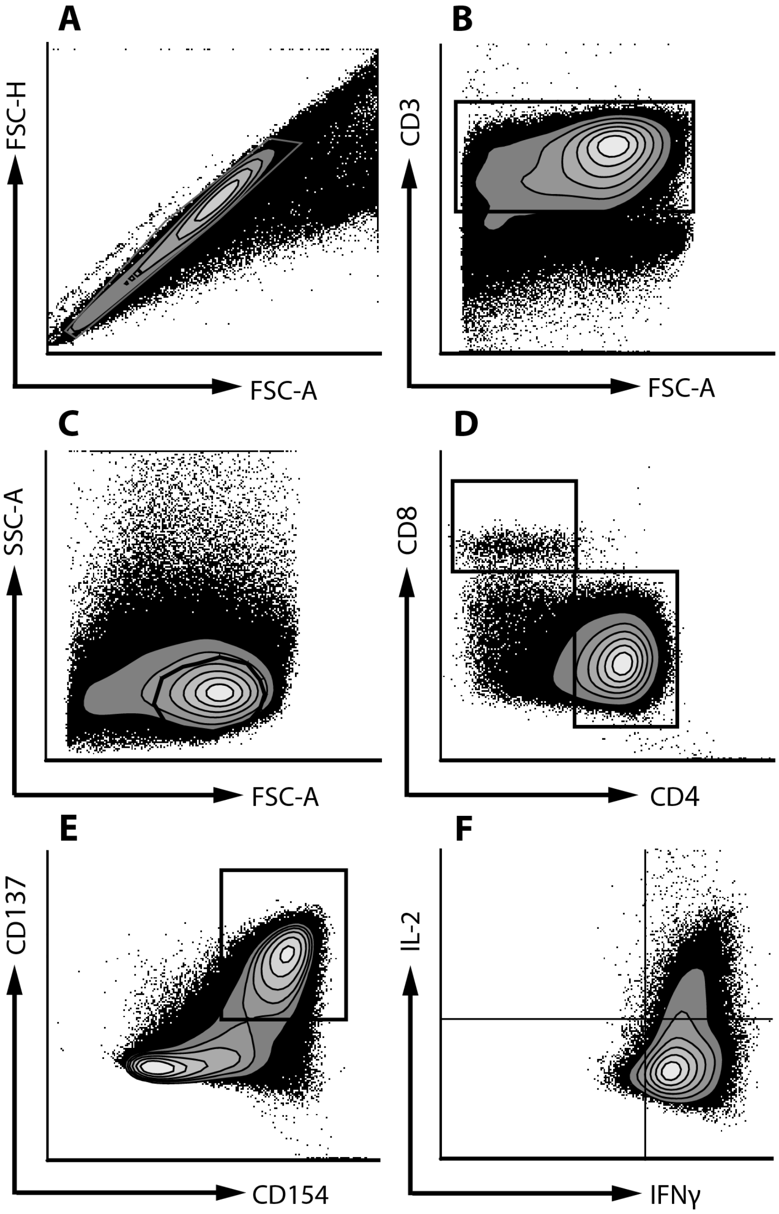

Legend Supplementary Figure 1:

First, gating is performed to include only single cells (singlets) (**A**) and CD3+ cells (**B**). After gating on the lymphocyte population (**C**), the CD4 and CD8 gates are set (**D**). The CD4 population is plotted for T cell activation markers CD137 and CD154 (**E**). A gate is set around the double positive population after which IFNγ and IL-2 upregulation within this population is plotted (**F**).

Supplementary Figure 2. XAGE-1b immunohistochemistry

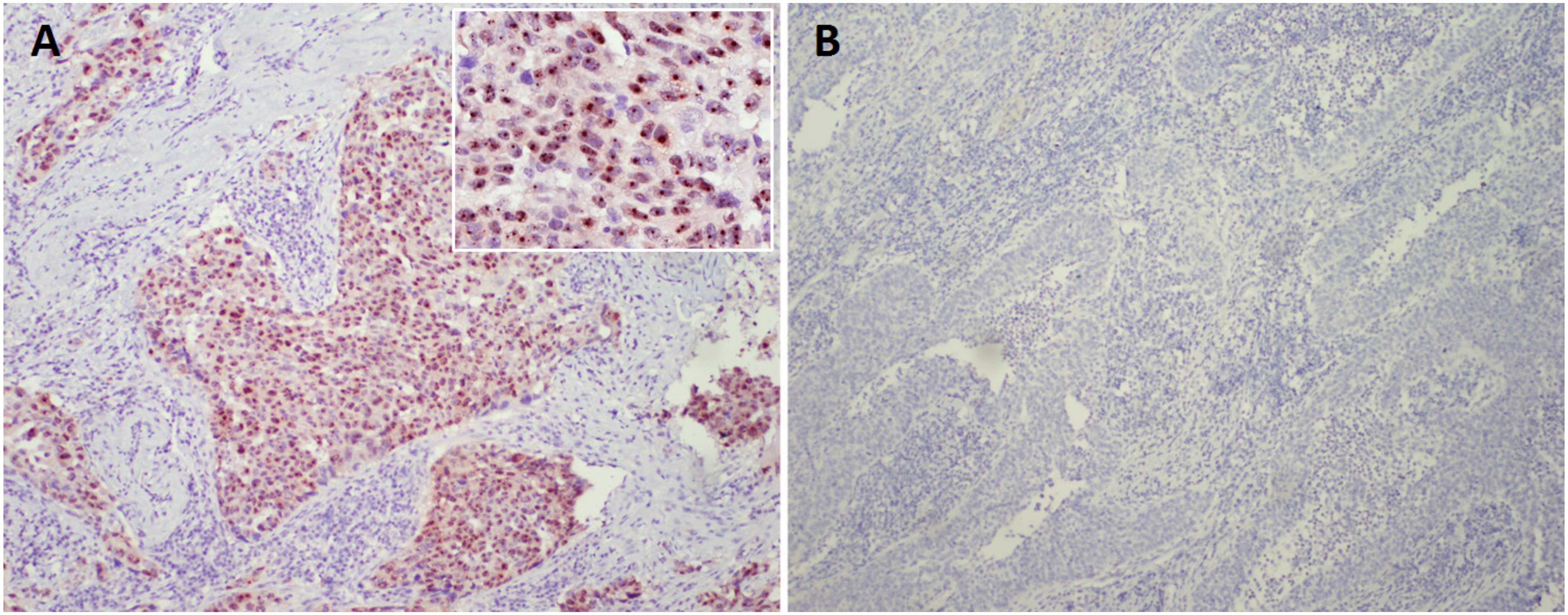

Legend Supplementary Figure 2:

Examples of **A)** a XAGE-1b positive tumor (patient X-14) with a diffuse (>50% tumor cells positive) staining pattern and **B)** a XAGE-1b negative tumor (patient X-18) at a magnification of 200x. The insert in **A)** is a digital magnification showing the typical nuclear staining of XAGE-1b in cancer cells.

Supplementary Figure 3. Frequency of T-cell phenotypes in peripheral blood, tumor and its draining lymph nodes

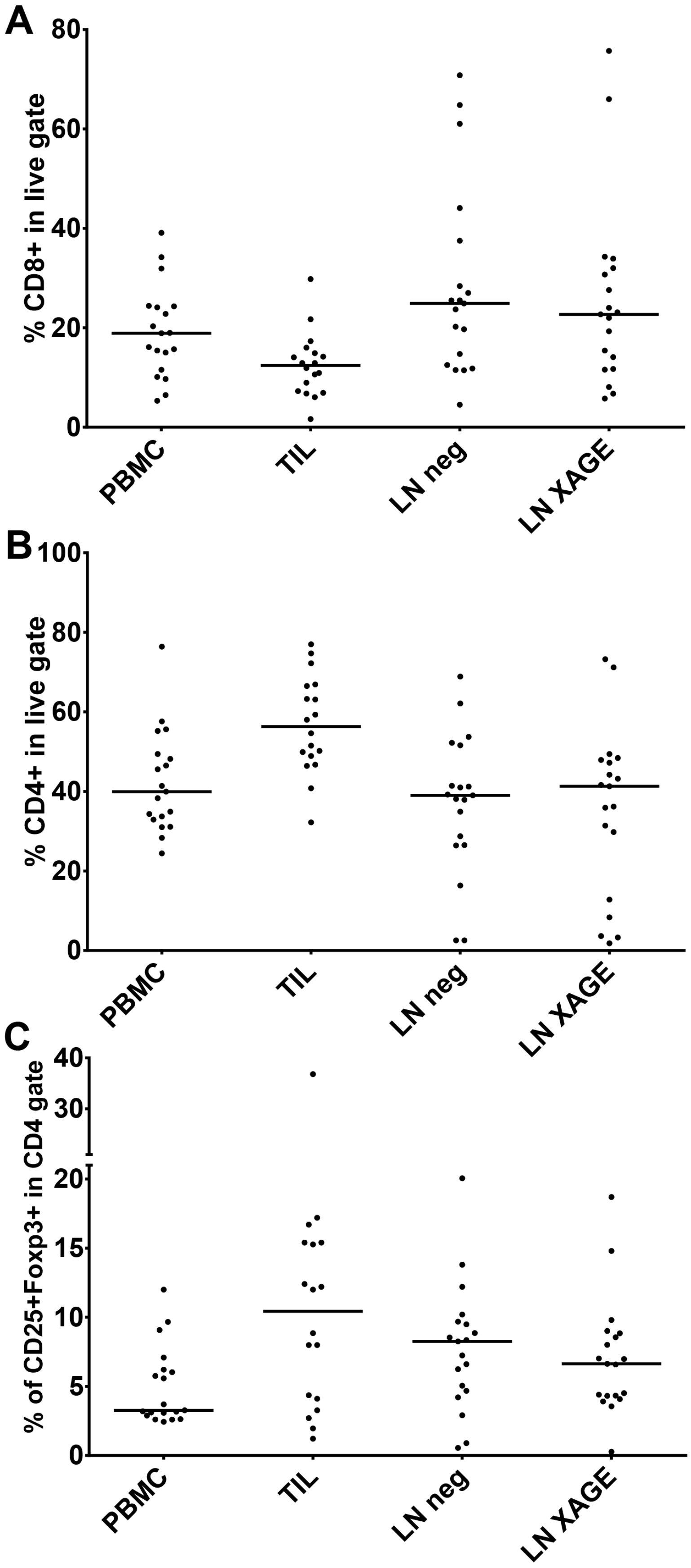

Legend Supplementary Figure 3:

T cells were analyzed by flow-cytometry after being stained for CD4, CD8, CD25 and Foxp3. T cells were derived from PBMC (direct ex-vivo), primary tumor (TIL, *in vitro* expanded) and draining lymph node (LN) expanded *in vitro* in the presence (LN XAGE) or absence (LN neg) of overlapping XAGE-1b peptides. Results from 20 adenocarcinoma patients are shown (dots) and the grand median is displayed (horizontal line).

A) $CD8^+$ and **B)** $CD4^+$ T-cell frequency in live cells. **C)** Frequency of $CD4^+CD25^+Foxp3^{high}$ T cells in PBMC, TIL and LN cells. A gating example is displayed in Supplementary Figure 1**.**

Supplementary Figure 4. Local XAGE-1b mediated immunity, Th2 response

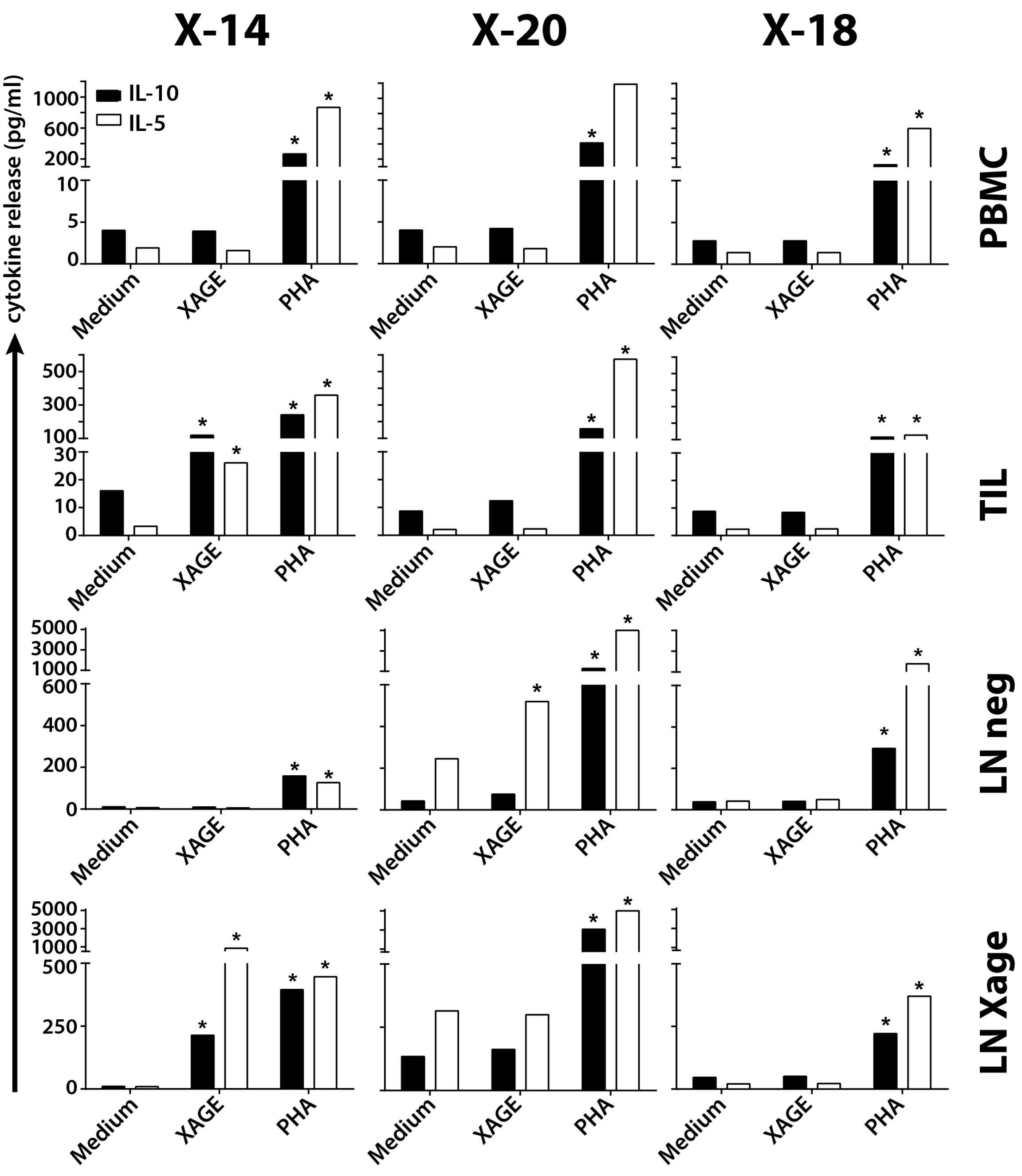

Legend Supplementary Figure 4:

Day 2 supernatants from PBMC, TIL and LN cells tested in a proliferation assay were analyzed for Th2 cytokine release (IL-10, IL-5). A positive response (indicated with asterisks) was defined by a cytokine concentration above the cut-off value (above 20 pg/mL) and more than twice the concentration of medium control. PHA was used as positive control. Results from two positive (X-14, X-20) and one negative (X-18) patients are shown.

Supplementary Figure 5: *Ex-vivo* XAGE-1b specific IFNγ response by ELISPOT assay

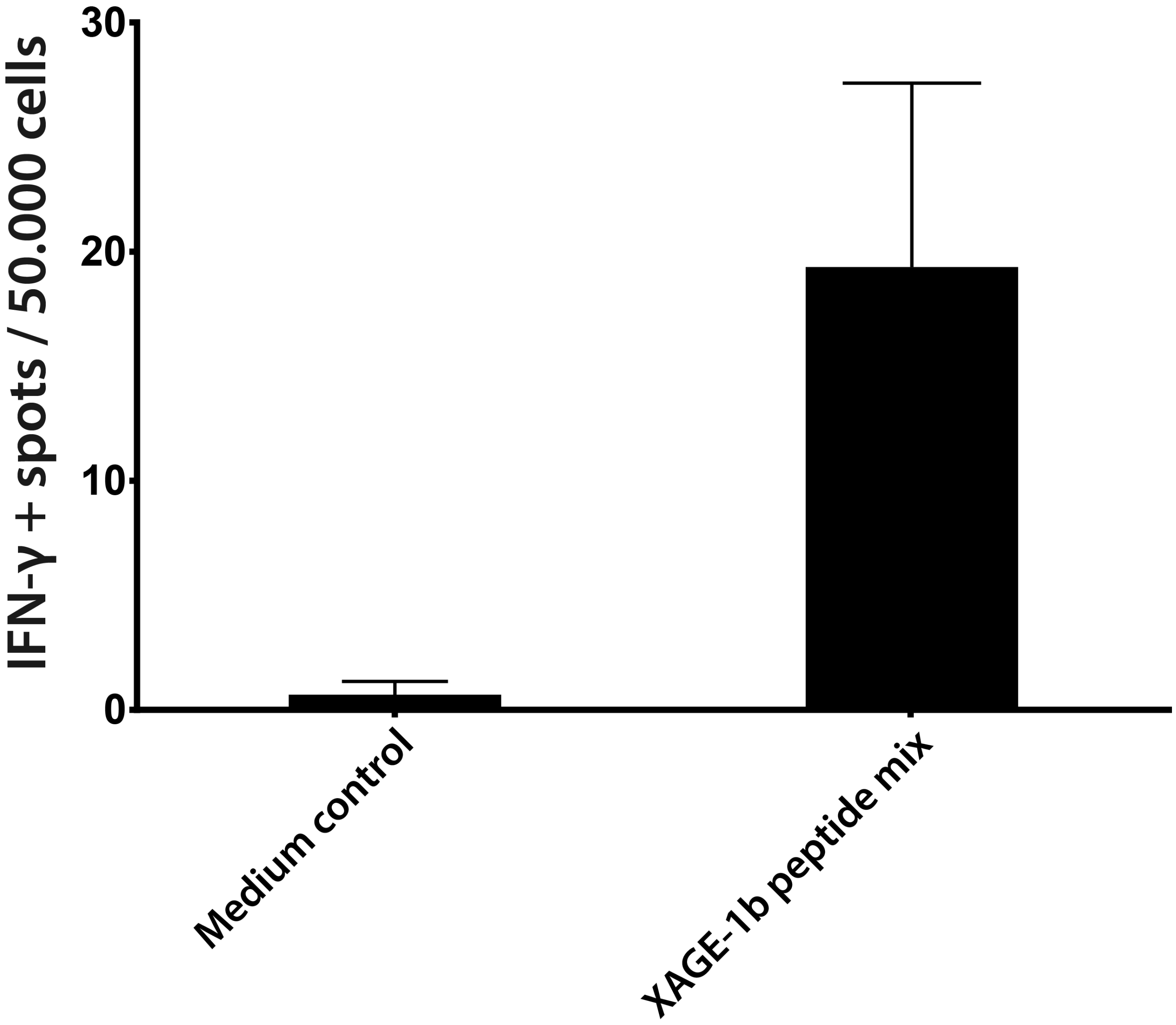

Legend Supplementary Figure 5:

PBMC samples of patient X-4 were evaluated for XAGE-1b reactivity in a direct ex-vivo ELISPOT assay as described in the Methods section. XAGE-1b specific T-cell frequencies were considered to be increased compared to medium control when frequencies are ≥ 1/10,000 cells.

Supplementary Figure 6. Circulating T cells show a XAGE-1b-specific response after removal of CD14+ cells

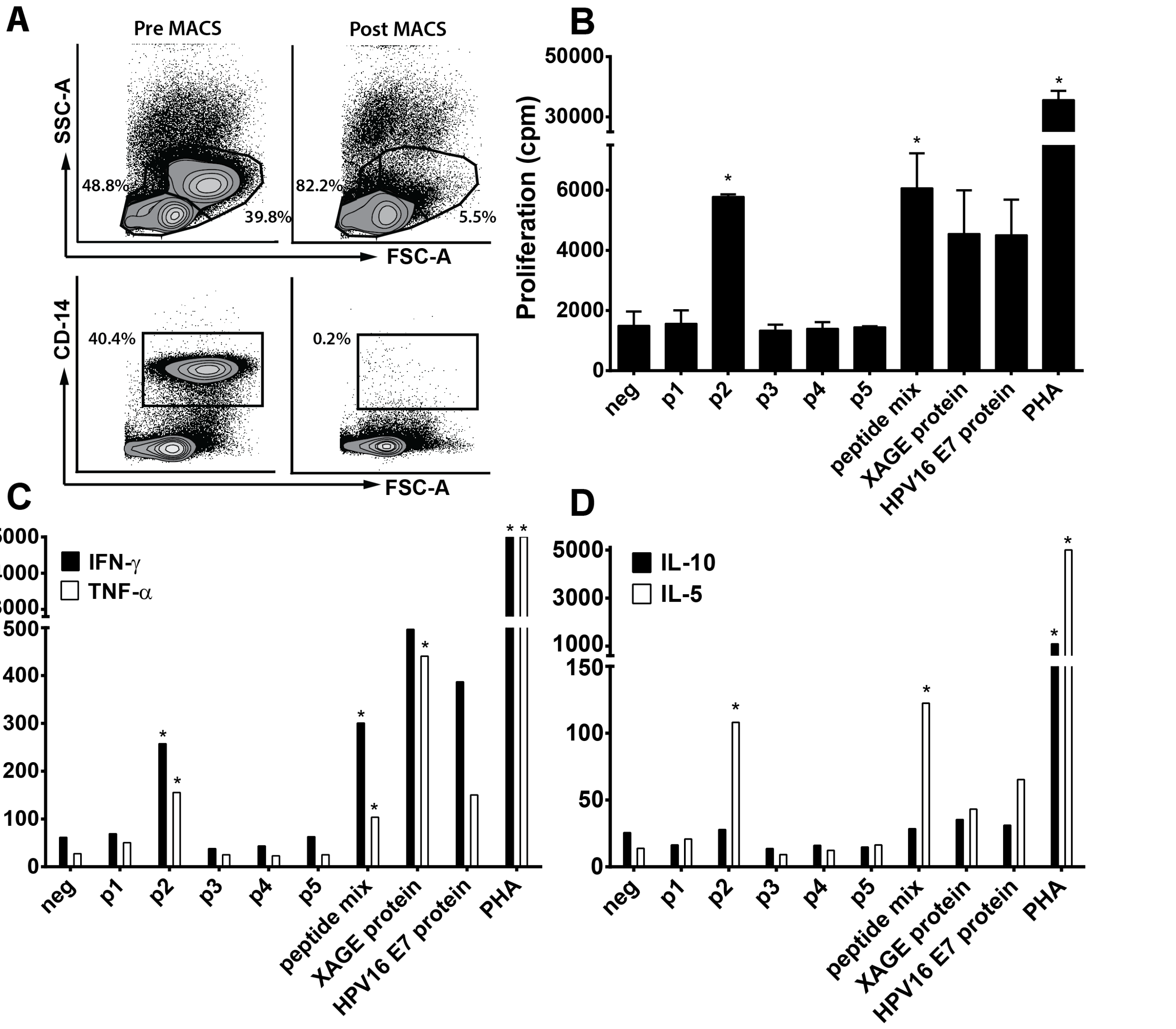

Legend Supplementary Figure 6:

A) Patient X-27 displayed a high frequency of myeloid cells in PBMCs (39.8%). CD14+ cells were removed from PBMCs by magnetic-activated cell sorting. The contour plots before and after sorting show the removal of the CD14+ cells (5.5% post-sorting) which were the main population of myeloid cells. **B)** The remaining cells were stimulated for 10 days with XAGE-1b overlapping peptides, after which XAGE-1b-specific proliferation was demonstrated for peptide p2 and XAGE-1b peptide mix. The supernatants of these cultures were analyzed for secretion of **C)** IFNγ and TNF-α and **D)** IL-5 and IL-10. Asterisks indicate a positive response.

Supplementary Figure 7. XAGE-1b-specific CD8 T cell response

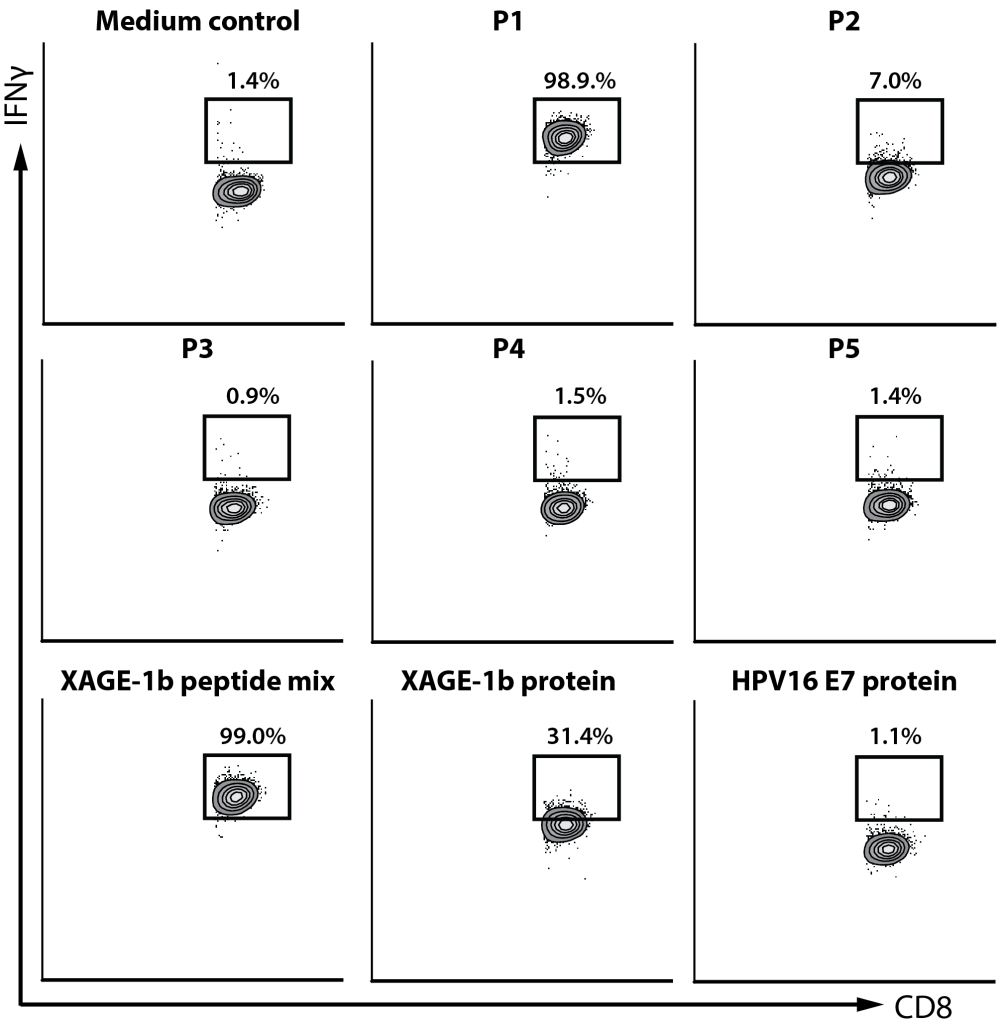

Legend Supplementary Figure 7:

The $CD8^+$ T cells in the 8 weeks cultured PBMCs of patient X-14 were gated (see Supplementary Figure 1). These expanded $CD8^+$ T cells showed an IFNγ response when stimulated with APCs pulsed with individual peptide p1, the peptide mix and to XAGE-1b protein, but did not react after stimulation with the other peptides or the negative control protein (HPV16 E7).

Supplementary Figure 8. Expression of T-cell activation marker CD137 in patient X-14

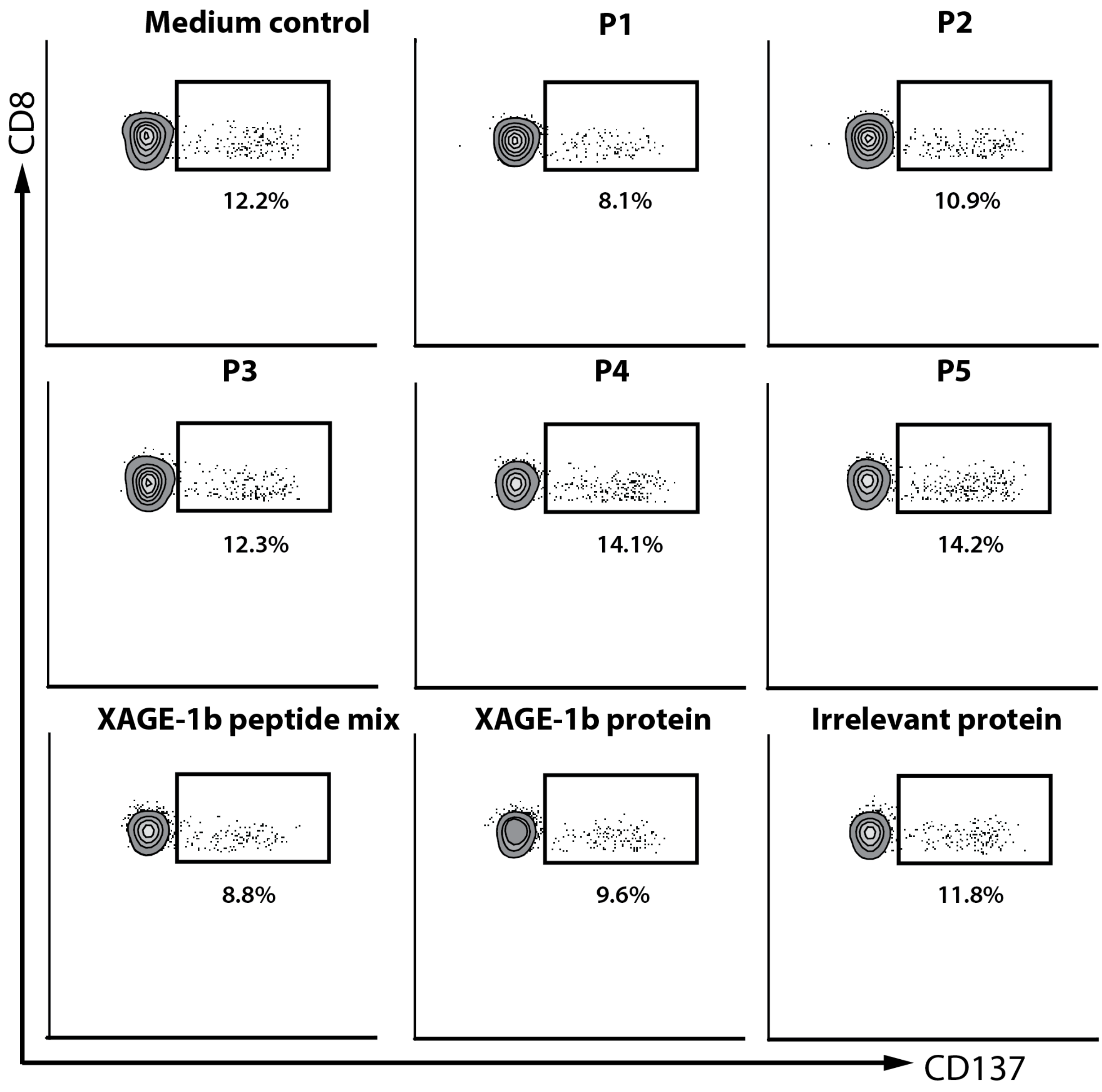

Legend Supplementary Figure 8:

The $CD8^+$ T cells in the 8 weeks cultured PBMC of patient X-14 were gated (see Supplementary Figure 1) and tested for XAGE-1b peptide (p1-p5) and protein specific expression of CD8 T-cell activation marker CD137. Although a peptide-specific intracellular IFNγ production was observed particularly for p1, peptide mix and XAGE-1b protein (see Supplementary Figure 7), this reaction was not accompanied by an upregulated expression of CD137.

Supplementary Figure 9. TCR-Vβ expression and XAGE-1b-specific proliferation of bulk cultured PBMC of patient X-4

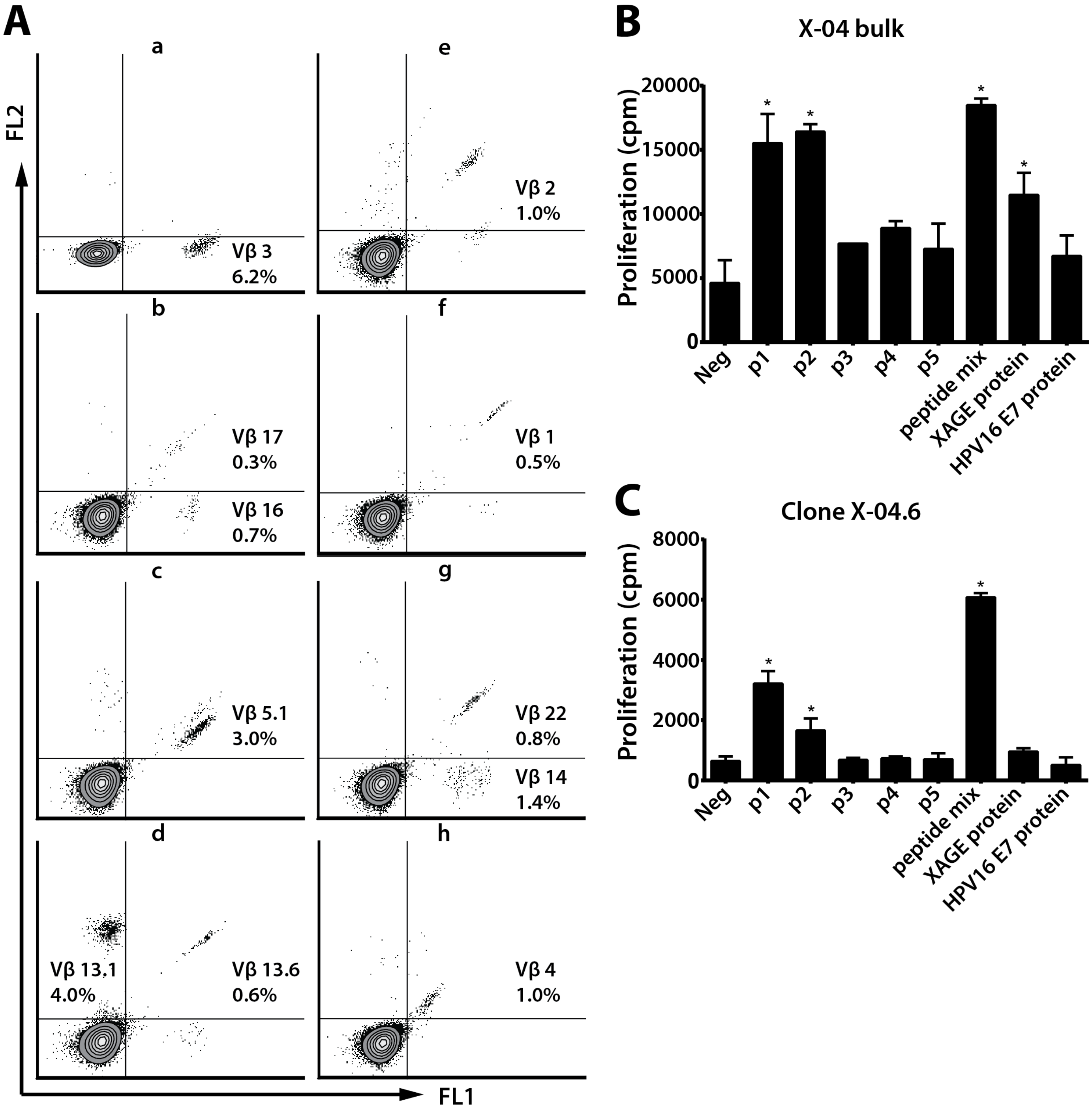

Legend Supplementary Figure 9:

A) Analysis of TCR-Vβ usage of bulk cultured PBMC of patient X-4. Expression was determined using eight sets of antibodies (a-h), each consisting of three differently labeled antibodies [FITC (FL1), PE (FL2), or FITC-PE combined] specific for 3 different TCR-Vβ families, ultimately covering about 70% of the human TCR-Vβ repertoire. Eleven different TCR-Vβ families (percentage of responding cells

indicated in the quadrants) were demonstrated in the expanded PBMCs that were kept in culture during cloning protocol. **B)** Proliferation assay of bulk cultured PBMCs of patient X-4 shows specific response to peptide p1, p2, the peptide mix and XAGE-1b protein. Asterisks indicate a positive response. **C)** Proliferation assay of clone X-4.6 shows a proliferation response to peptide p1, p2 and the peptide mix. Asterisks indicate a positive response.
